# Adaptive Neuro-Fuzzy Inference System Based Orientation Control of an Intra-operative Ultrasound Robot

**DOI:** 10.1088/1757-899X/470/1/012031

**Published:** 2019-01-24

**Authors:** Shuangyi Wang, James Housden, Davinder Singh, Kawal Rhode

**Affiliations:** 1 School of Biomedical Engineering & Imaging Sciences, King’s College London; 2 Xtronics Ltd., Gravesend, Kent, UK

## Abstract

Trans-esophageal echocardiography (TEE) is a miniatured intra-operative ultrasound system, widely used in routine diagnosis and interventional procedure monitoring, to assess cardiac structures and functions. As a way to assist the operation of TEE remotely, we have developed an add-on robotic system to actuate a commercial TEE probe. For the proposed robot, understanding the inverse kinematics (IK) which relates the probe pose to the joint parameters is the fundamental step towards automatic control of the system. Rather than using conventional numerical-based techniques which may have problems with speed, convergence, and stability when applying to the TEE robot, this paper explores a soft computing approach by constructing an Adaptive Neuro-Fuzzy Inference System (ANFIS) to learn from training data generated by the forward kinematics (FK) and then computing the inverse kinematics in order to control the orientation of the TEE probe. With 1900 training data over 40 epochs, the minimum training error for each joint parameter was found to be less than 0.1 degree. Validation using a separate data set has indicated that the maximum error was less than 0.3 degree for each joint parameter. It is therefore concluded that the ANFIS-based approach is an effective way, with acceptable accuracy, to compute the inverse kinematics of the TEE robot.

## 1 Introduction

Trans-esophageal echocardiography (TEE) is an important type of intra-operative ultrasound system that has been widely utilized for cardiac disease diagnosis and interventional procedure guidance [1, 2]. TEE uses a cable actuated steerable probe system, in which the bi-directional bending of the distal rigid end of the probe in a bending plane is controlled by two independently rotatable control knobs on the handle of the system. The ultrasound transducer is embedded into the distal rigid end of the probe, providing unique features of this probe for imaging and assessing heart structure and function via esophagus. To facilitate the use of TEE system, we have developed the first add-on robot [3] working with a commercial TEE probe to enable remote control of the system, and therefore keeping the operator (known as echocardiographer) away from radiation exposure when TEE is used in conjunction with X-ray system during interventional procedure guidance. The robotic system has four degrees of freedom (DOFs) mechanically, including the translation of the probe along the esophagus, rotation of the probe about the long axis of the handle, and bi-directional bending of the probe tip at the distal end of the robot-probe system, similar to the manual control of the probe.

Working towards automation, understanding the kinematics of the TEE robot is a fundamental step. In our previous work, a model for forward kinematics (FK), which relates the joint parameters to the location of the probe tip, i.e., its position and orientation, was descripted and validated experimentally. While the forward kinematic model is established, the inverse kinematics (IK) problem, which aims to calculate the joint parameters when giving a target probe tip location, is found to be a challenging task, since the mapping between Cartesian space and joint space is non-linear in nature and involves redundancy problem with multiple solutions caused by the combination use of handle rotation axis and bi-directional bending axes of the TEE robot in adjusting the orientation of the probe tip.

While the exact IK solution of the TEE robot is seldom covered in the literature, several existing works have been presented for the similar types of robot where the actuated device is similar to the TEE probe mechanically, such as the intra-cardiac ultrasound catheter robot [4], the endoscopic robot for natural orifice transluminal surgery [5], and the cardiac ablation catheter robot [6]. Among these works, the most common approach for solving the IK problem is to use Jacobian-based inverse technique [7], which uses the iterative optimization based on the differential changes between the joint parameters and the end-effector’s location in the Cartesian space. In addition to these works, we also explored the gradient-descent search scheme in solving the IK problem for the TEE robot in our previous work described in [8]. Although these methods have demonstrated successful solution to the IK problem, numerical-based iteration methods are time consuming and do not guarantee convergence to an accurate solution.

In this paper, we aim to explore a soft computing approach by constructing an Adaptive Neuro-Fuzzy Inference System (ANFIS) [9–11] to learn from training data generated by the FK of the TEE robot and then compute the IK using the trained system. With the recent works using ANFIS to test the arm-based robotic systems, such as described in [12–16], this approach has been proved to be an efficient way in solving IK problem and would provide a faster solution without the need of numerical iterations in real-time application after the network is trained. Since the use of ANFIS to solve the IK problem for the endoscopic robot has not been clearly reported, the objective of this paper is to test the effectiveness and accuracy of the method in solving IK problem for the TEE robot, which also provides the feasibility of using such method in solving other similar types of endoscopic robots.

## 2 FK of the TEE robot

**Figure 1:**
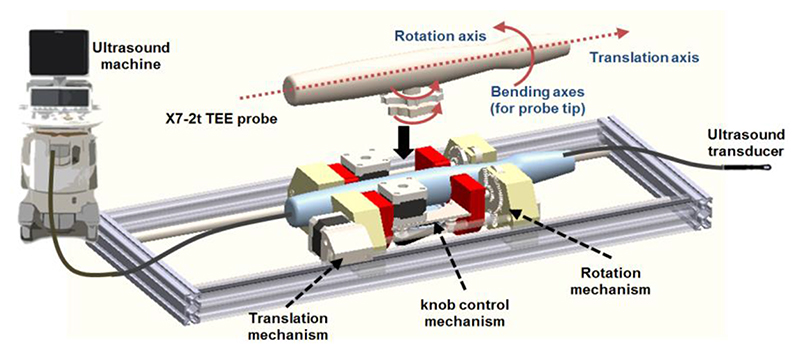
Overview of the proposed TEE robot with the original probe and moving axes shown.

The developed add-on TEE robot (Figure 1) holds and manipulates the probe handle with a commercial TEE probe (x7-2t, Philips, Amsterdam, The Netherlands) plugged into the robot. The system comprises three structures [3]: the handle control structure to rotate the two knobs in an actuating chamber; a probe rotation mechanism for rotating the shaft about the long axis of the probe; and a linear belt mechanism mounted on a rail to provide translation movement.

**Figure 2:**
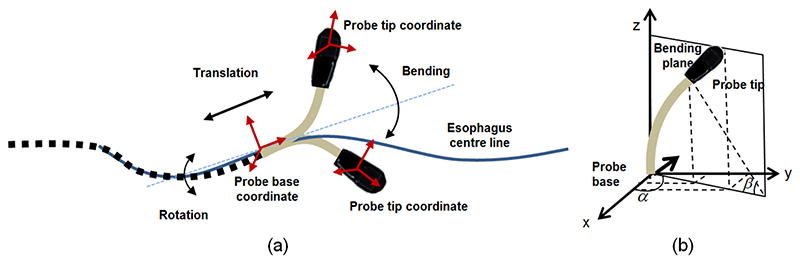
(a) Overall geometric illustration of the movements of the TEE robot modelled in the FK. (b) The projection view in the bending plane for the bi-directional bending axes.

With the translation of the probe modelled by an interpolation of the esophagus’s centre line, the probe base coordinates could be easily decided mathematically. The FK then models rotational movements of the TOE probe handle with the rotation angle *v,* which can be described by a standard rigid transformation. The bi-directional bending of the probe tip has been previously described in [3]. The inputs are the rotation angle of the first bending knob, *φ_x_,* which controls the bending tip pitch in the posterior-anterior plane; and the rotation angle of the second bending knob, *φ_y_,* which controls the bending tip yaw in the left-right plane. The geometric illustration of the FK of the TEE robot is shown in Figure 2. The transformation matrix between the probe tip coordinates and the probe base coordinates, after translation of the probe, is expressed as:

(1) $$\text{T}=\left[\begin{array}{cccc} C_{t}(C_{\beta }C_{\alpha }^{2}+S_{\alpha }^{2})-C_{\alpha }S_{\alpha }S_{t}(C_{\beta }-1) & C_{\alpha }S_{\alpha }C_{t}(C_{\beta }-1)-S_{t}(C_{\alpha }^{2}+C_{\beta }S_{\alpha }^{2}) & C_{\alpha }S_{\beta }C_{t}-S_{\alpha }S_{\beta }S_{t} & C_{t}(L_{u}C_{\alpha }S_{\beta }-(L_{f}C_{\alpha }(C_{\beta }-1)/\beta )-S_{t}(L_{u}S_{\alpha }S_{\beta }-(L_{f}S_{\alpha }(C_{\beta }-1)/\beta )\\ S_{t}(C_{\beta }C_{\alpha }^{2}+S_{\alpha }^{2})+C_{\alpha }S_{\alpha }C_{t}(C_{\beta }-1) & C_{\alpha }S_{\alpha }S_{t}(C_{\beta }-1)+C_{t}(C_{\alpha }^{2}+C_{\beta }S_{\alpha }^{2}) & C_{\alpha }S_{\beta }S_{t}+S_{\alpha }S_{\beta }C_{t} & S_{t}(L_{u}C_{\alpha }S_{\beta }-(L_{f}C_{\alpha }(C_{\beta }-1)/\beta )+C_{t}(L_{u}S_{\alpha }S_{\beta }-(L_{f}S_{\alpha }(C_{\beta }-1)/\beta )\\ -C_{\alpha }S_{\beta } & -S_{\alpha }S_{\beta } & C_{\beta } & L_{u}C_{\beta }+(L_{f}S_{\beta }/\beta )\\ 0 & 0 & 0 & 1 \end{array}\right]$$

where α is the angle between the bending plane and the X-Z plane, β is the bending angle in the bending plane, L_*f*_ is the length of the bending section of the probe tip, and L_*u*_ is the length of the rigid section of the probe tip. In the matrix, *Sx* and *Cx,* respectively, denote *sin(x)* and *cos*(*x*).

## 3 Proposed ANFIS architecture

ANFS is a combination of two soft-computing methods of artificial neural network (ANN) and fuzzy inference system (FIS), which constructs an adaptive network that uses supervised learning on learning algorithm. By using ANN to automatically adjust parameters of membership functions (MFs) of fuzzy inference systems, the network brings learning capabilities of neural networks to fuzzy inference systems. Using ANFIS, the inputs are mapped through input MFs and associated parameters, and then through output MPs and associated parameters to outputs. The parameters associated with the MFs changes through the learning process. The computation of these parameters is facilitated by back propagation algorithm alone or in combination with a least squares type of method. The structures of the ANFIS network can be divided into fuzzification, inference, implication, and aggregation:

- *Fuzzification:* Every node in this layer adapts to a function parameter. The output from each node is a degree of membership value that is given by the input of the membership functions.
- *Inference:* Every node in this layer is a fixed or non-adaptive node. In each node, the result of multiplying of signal coming into the node is firstly calculated, which represents the firing strength for each rule. Then the ratio between the i-th rules firing strength and the sum of all rules’ firing strengths, known as the normalized firing strength, is the output from each node.
- *Implication*: Every node in this layer is an adaptive node to an output. The output from each node is decided by the output membership function.
- *Aggregation*: The single node in this layer is a fixed or non-adaptive node that computes the overall output as the summation of all incoming signals from the previous node.

**Figure 3:**
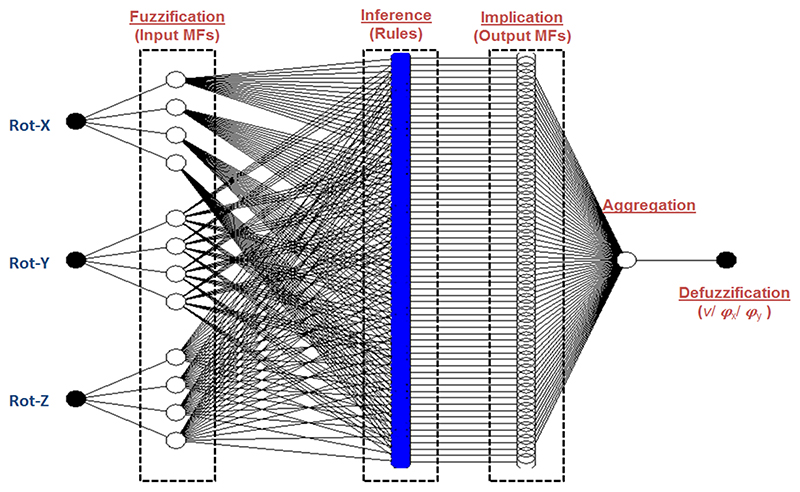
Proposed ANFIS architecture for computing the IK problem of the TEE robot.

In the specific application of TEE, where the probe is constrained by the esophagus (less than 2 cm in diameter) and orientated towards the heart for ultrasound imaging, the translation movement along the esophagus can be easily decoupled and compensated from the kinematics model mathematically. As for the remaining Cartesian parameters of the probe tip location: Trans-X, Trans-Y, Rot-X, Rot-Y, and Rot-Z, the control of the orientation of the probe tip is important for visualizing the heart, while the translation offsets due to the orientation change are small and have less impacts to the ultrasound visualization with the constraint from the esophagus. Therefore, the inputs of the considered ANFIS network are set to be the three rotation angles representing the orientation of the probe tip and the outputs are the joint parameters, the handle rotation angle *v* and the rotation angle of the bending knobs *φ_x_* and *φ_y_*. In total, the ANFIS architecture consists of three ANFIS networks, with one for each joint parameter. In the network, four input membership functions are utilized based on our experimental trials. Accordingly, 64 numbers of fuzzy rules and output membership functions are constructed. The structure of the considered ANFIS network is shown in Figure 3.

## 4 Training and validation

**Figure 4:**
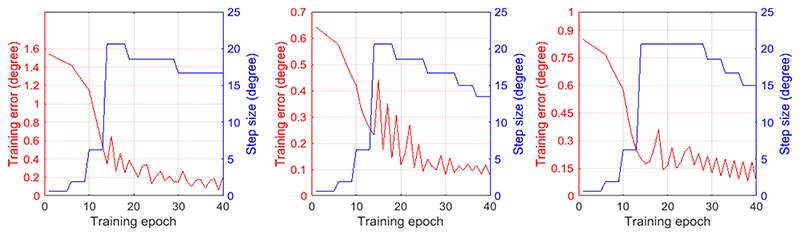
Change of training error (in red) and step size (in blue) over training epochs for each ANFIS network. From left to right: probe axial rotation, probe left-right bending, and probe up-down bending.

Using the FK solution described by formula (1), training data (1900 sets) are generated for different combinations of the robot joint parameters (axial handle rotation angle and bi-directional bending angles) at certain intervals within the working space and the resulted probe tip orientation (three decomposed rotation angles). After our experimental trials, 40 training epochs were selected for training of each ANFIS network. The change of the training error after each epoch is shown in Figure 4. The step size for the training process of each network has been tuned to an optimal profile (Figure 4), which increases initially, reach a maximum, and then decrease for the rest of the training.

**Figure 5:**
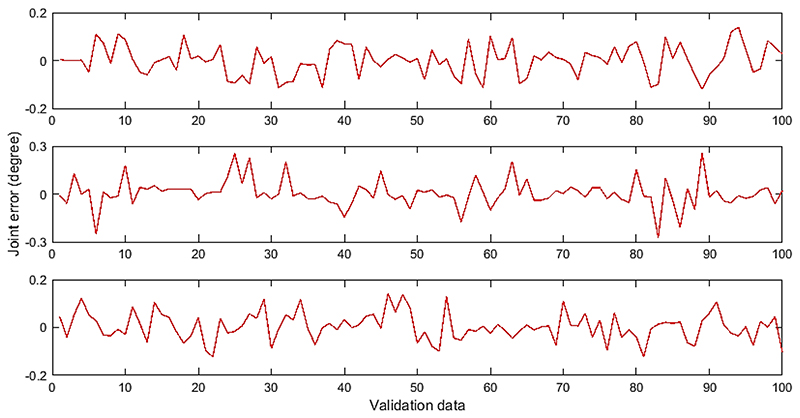
Joint errors for each ANFIS network validated using a separate data set. From top to bottom: probe axial rotation, probe left-right bending, and probe up-down bending.

To validate the performance of the ANFIS-based IK after each network is trained, a separate validation data set is created using the FK model with 100 randomly generated probe tip locations within the workspace. The according joint parameters were recorded and treated as the desired outputs of the IK solution. The three decomposed rotation angles were fed into each ANFIS network and the combined outputs will result in a set of output joint parameter. The differences between the desired joint parameters and the resulted output joint parameter from the ANFIS networks are treated as the errors of the proposed method, which are summarized in Figure 5.

## 5 Discussion and Conclusion

This paper introduces an ANFIS-based IK solution of a newly developed TEE robot, aiming at control the orientation of the TEE probe to visualize the heart. Three ANFIS networks were established with orientation of the probe tip as the input and the three joint parameters as the outputs. The networks were successfully trained using the data set generated by the FK solution with errors converged to the values less than 0.1 degree. A separate randomly generated data set was used for validation with maximum errors found to be less than 0.3 degree for all the joint parameters. The ANFIS-based IK solution has shown a reliable performance with an acceptable error range. Compared with the conventional numerical-based IK solutions, the ANFIS-based soft computing approach do not require heavy interactive computing for calculation of the joint parameters once the networks are trained. Therefore, such solution would be ideal for real-time applications, such as the feedback control to automatically adjust the TEE probe in order to keep track of a moving structures or interventional devices. Compared with geometry-based analytical IK solutions which are usually difficult to derive, the ANFIS-based solution is easy to implement with the training data generated using the FK, thus providing a more generic solution. This paper has verified the effeteness and accuracy for the ANFIS-based IK method used for a TEE robot. Considering the similar mechanical configuration, it is believed that several other types of endoscopic robots could use the same solution for calculating IK.

Based on the results from the training and the validation experiments, it is concluded that the ANFIS-based IK solution is feasible and accurate for control the orientation of the TEE robot. Future works will focus on performing more task-driven experiments to further validate the method and also incorporating the additional DOF of ultrasound image steering controlled by the ultrasound system into the ANFIS networks to provide an extended IK solution to the image space.
